# Retinoic Acid-Related Orphan Receptor Alpha May Regulate the State of Hair Follicle Stem Cells by Upregulating the Expression of BNIP3

**DOI:** 10.3390/ani14233477

**Published:** 2024-12-02

**Authors:** Yu Zhang, Xuefei Zhao, Shuqi Li, Yanchun Xu, Suying Bai, Wei Zhang

**Affiliations:** 1 College of Wildlife and Protected Area, Northeast Forestry University, Harbin 150040, China; zhangyunefu@163.com (Y.Z.); xu_daniel@163.com (Y.X.); 2 National Forestry and Grassland Administration Research Center of Engineering Technology for Wildlife Conservation and Utilization, Harbin 150040, China; 3 Detecting Center of Wildlife, State Forestry and Grassland Administration, Harbin 150040, China

**Keywords:** hair follicle stem cells, retinoic acid-related orphan receptor alpha, *Bnip3*

## Abstract

The hair, an exclusive keratinized dermal appendage in mammals, stands as a quintessential outcome of adaptive evolution, conferring resilience against adverse environmental conditions. The ontogenesis of the coat displays a pronounced rhythmic pattern, with hair follicle stem cells (HFSCs) emerging as pivotal facilitators of hair follicle reconstitution. The retinoic acid-related orphan receptor alpha, a nuclear receptor with extensive involvement in the regulation of cellular physiological states, exerts its functions predominantly through the modulation of downstream target gene transcription. The *Bnip3* gene exhibits a robust correlation with cellular apoptosis and autophagy, which are indispensable physiological mechanisms underlying the maintenance of HFSC homeostasis. Consequently, the expression level of *Bnip3* may be intimately linked to the status of HFSCs. In this investigative endeavor, we employed rat HFSCs as a model system to validate the regulatory impact of RORA on *Bnip3* gene expression. Our findings unequivocally demonstrate that *Bnip3* serves as a direct downstream target of RORA. Specifically, RORA binds to the motif within the *Bnip3* promoter region, thereby upregulating *Bnip3* expression levels. In light of our research findings, we propose that RORA holds potential as a target for modulating the status of HFSCs.

## Simple Summary

This study demonstrates that *Bnip3* is a downstream target gene of the retinoic acid-related orphan receptor alpha (RORA), and RORA upregulates the transcription level of *Bnip3* by binding to a motif in the *Bnip3* promoter region. This regulatory relationship may have the potential to maintain the homeostasis of hair follicle stem cells.

## 1. Introduction

The hair, a distinctive epidermal keratinized structure in mammals, fulfills multiple roles, including protection, thermal insulation, sensation, and the manifestation of secondary sexual characteristics. It represents a quintessential outcome of adaptive evolution [1,2,3]. Hair follicles are pouch-like tissues formed by the invagination of the skin, and along with the enclosed hair roots and other structures, they constitute the basic structural units of animals’ hair [4,5]. A typical hair follicle comprises structures such as the hair bulb, hair shaft, root sheath, arrector pili muscle, and sebaceous gland. Approximately dozens of cell types participate in the construction of this minute organ, the hair follicle [6]. It is noteworthy that hair follicle development exhibits a typical cyclical nature, which can be broadly classified into anagen, catagen, and telogen based on the degree of its developmental activity [7,8,9]. In addition to its intrinsic developmental cyclicity, the hair of many animals also displays a distinct seasonal rhythm [10,11].

Hair follicle stem cells (HFSCs), residing in the bulge area at the junction of the arrector pili muscle and the outer root sheath, are typical adult stem cells possessing the potential for self-renewal and multilineage differentiation, and they are the most crucial cell type in the process of hair follicle development [12,13,14,15,16]. During the catagen and telogen phases of hair follicle development, hair follicle stem cells exhibit a slow cycling behavior. When the hair follicle enters the anagen phase, these stem cells rapidly proliferate, differentiate, and migrate, providing new cells for the growing hair follicle and maintaining its normal structure and function [17]. Furthermore, when skin tissue is damaged, hair follicle stem cells can migrate to the injured site and execute repair functions [18,19,20]. Most hair follicles undergo lifelong cyclical development, and thus, an adequate reserve of hair follicle stem cells is a crucial factor in maintaining this prolonged physiological process. Existing research indicates that the loss or even depletion of the stem cell pool due to an abnormal migration of hair follicle stem cells during aging may be a significant cause of hair loss in the elderly [21]. There are also studies that have found that hair follicle stem cells seem to possess the ability to perceive their own state; through a complex regulatory network of genes such as *Foxc1* (Forkhead Box C1), these cells can timely switch active hair follicle stem cells to a quiescent state, thereby preventing them from being excessively consumed during the anagen phase of hair follicle growth [22].

The *Bcl-2* (B-cell lymphoma-2) family constitutes a gene family highly associated with cellular apoptosis, particularly playing a crucial role in apoptosis that relies on changes in mitochondrial membrane permeability. The *Bnip3* gene (BCL2 Interacting Protein 3) was also previously known as *Nip3*; although it is classified as part of the *Bcl-2* family, it only contains the BH3 domain (BCL-2 homology 3). Current research generally believes that this gene is involved in the occurrence of cell apoptosis and mitochondrial autophagy [23]. Since hair follicles are highly dependent on mechanisms such as apoptosis and autophagy to maintain their structure and homeostasis during the catagen and telogen phases, *Bnip3* may play a crucial regulatory role at these stages.

The retinoic acid-related orphan receptor alpha is a nuclear receptor widely distributed in various tissues of mammals. Its structure comprises four domains: the N-terminal region, DNA binding domain, hinge region, and ligand binding domain. It can recognize and bind to target gene-specific DNA response elements in the form of monomers or dimers, thereby regulating the transcription levels of downstream genes [24,25,26]. Previous studies have shown that *RORA* is broadly involved in cell proliferation, differentiation, migration, adhesion, and metabolic regulation. Additionally, it plays a significant role in the control of biological rhythms [27,28,29,30]. In studies related to hair follicle development, scholars have discovered that *RORA* may be highly involved in the periodic reconstruction and seasonal developmental regulation of hair follicles [31,32]. Functional studies of RORA have mostly concentrated on circadian rhythm-related directions, with relatively few reports on seasonal rhythm-related directions [33,34]. Previous studies have reported (with some controversy) that RORA is a natural receptor for melatonin. Melatonin, an important bridge medium linking photoperiod signals to adaptive physiological regulation in animals, is highly correlated with seasonal rhythmic changes in animals [35,36,37,38]. Although studies have shown the importance of RORA in regulating hair follicle development, it is still unclear whether RORA can affect the state of HFSCs and the underlying molecular mechanisms from the perspective of apoptosis. In previous studies, we noticed that RORA seems to have a regulatory relationship with the *Bnip3* involved in apoptosis regulation. Therefore, this study attempts to verify the regulatory effect between the two and discuss the potential impact of RORA on HFSCs from the perspective of apoptosis.

## 2. Materials and Methods

### 2.1. Cell Culture and Drug Treatment

The acquisition of primary hair follicle stem cells derived from Sprague Dawley rats and the culture conditions used in this study refers to the reports by Oshima, Rochat, and Shwartz et al. [39,40,41]. Following a 24 h adherence and equilibration period, the HFSCs were subjected to treatment with the RORA agonist SR1078 (MCE, HY-14422) at a final concentration of 10 μM for a duration of 24 h. A DMSO-treated control group was concurrently established to serve as a benchmark for comparison.

### 2.2. RNA Isolation and Reverse Transcription

After treating the cells with the drug for 24 h, the medium was removed, and the cells were washed three times with PBS buffer. The FastPure Cell/Tissue Total RNA Isolation Kit V2 (Vazyme, RC112-01, Nanjing, China) was utilized for RNA purification. For the synthesis of the first strand of cDNA, the PrimeScript™ RT reagent Kit with gDNA Eraser (Takara, RR047A, Kusatsu, Japan) was employed, following the protocol outlined in the kit’s instruction manual.

### 2.3. Quantitative Real-Time PCR (qRT-PCR)

The SsoAdvanced™ Universal SYBR^®^ Green (Bio-Rad, 1725270, Hercules, CA, USA) and the Bio-Rad CFX384 Real-Time PCR Detection System were used for real-time quantitative PCR detection of gene differential expression. The reaction system and program were set according to the kit’s instruction manual, with the melting curve analysis conducted using the default program of the instrument. The primer sequences are provided in the Supplementary Materials. The gene *Ppib* (Peptidylprolyl Isomerase B) was used for normalization, and the 2^−ΔΔCt^ method was employed for differential expression analysis.

### 2.4. Total Protein and Nuclear Protein Isolation

HFSCs treated with drugs were digested using 0.25% trypsin (Gibco, 25200056, Thermo Fisher, Waltham, MA, USA) that had been prewarmed to 37 °C. After digestion was terminated with serum-containing medium, the cell suspension was transferred to a centrifuge tube and centrifuged at 200× *g* for 5 min to obtain a cell precipitate. The cells were then resuspended in RIPA (Radio Immunoprecipitation Assay Lysis buffer) containing a final concentration of 1 mM PMSF (phenylmethanesulfonylfluoride or phenylmethylsulphonyl fluoride), vortexed thoroughly for lysis, and allowed to sit on ice for 10 min. Centrifugation was performed to obtain the supernatant, which contained the total cellular protein. For the extraction of nuclear proteins, the NE-PER Nuclear and Cytoplasmic Extraction Reagents (Thermo Fisher, 78833, Waltham, MA, USA) were used, following the protocol outlined in the kit’s instruction manual.

### 2.5. Western Blot

Protein samples were assayed for concentration employing the BCA method, and an equivalent volume of 4× Protein SDS-PAGE Loading Buffer (Takara, 9173, Kusatsu, Japan) was added to each. These samples were subsequently incubated at 99 °C for 10 min to ensure the complete denaturation of the proteins. A gradient SDS-PAGE gel, ranging from 4% to 20% (Genscript, M00655, Nanjing, China), served as the medium for protein separation via electrophoresis. Postelectrophoresis, the proteins were transferred onto a PVDF membrane. Prior to antibody incubation, the membrane underwent blocking with 5% BSA (dissolved in TBST) for 2 h. This was followed by an overnight incubation with primary antibodies of PPIB and *Bnip3* sourced from Proteintech (11607-1-AP and 68091-1-Ig). Subsequently, the primary antibodies were removed, and the membrane was washed thoroughly three times with TBST buffer. Then, the membrane was incubated with secondary antibodies for 1 h. After another set of washes, the membrane was subjected to ECL for visualization. Imaging was conducted using the Bio-Rad ChemiDoc MP Imaging System, and the grayscale intensities of the bands were analyzed using ImageJ software version 1.54k (National Institutes of Health, Bethesda, MD, USA).

### 2.6. Immunofluorescence

First, cell slides were prepared. After removing the cell culture medium, the cells were washed three times with PBS buffer. Acetone was then added to fix the cells for 15 min. The cells were permeabilized with 0.5% Triton X-100 at room temperature for 20 min, followed by blocking with goat serum for 30 min. Primary antibodies of *Bnip3* (Proteintech, 68091-1-Ig, Rosemont, IL, USA) were added and incubated at 4 °C overnight. After removing the primary antibodies and washing the cells, secondary antibodies were added and incubated at room temperature for 1 h. After another round of washing, a mounting medium containing DAPI anti-fade reagent was applied dropwise onto the cell slides. The slides were then transferred to a fluorescence microscope for image acquisition.

### 2.7. Cleavage Under Targets and Release Using Nuclease

HFSCs treated with SR1078 for 24 h were utilized for the Cleavage Under Targets and Release Using Nuclease (CUT&RUN) assay, with an IgG control group set up for comparison. The CUT&RUN Assay Kit (Cell Signaling Technology, #86652, Danvers, MA, USA) and DNA Purification Kit (Cell Signaling Technology, #14209) were used to prepare enriched DNA samples of RORA target gene binding regions, following the manufacturer’s instructions. Droplet Digital PCR (ddPCR) was then employed to detect the presence of *Bnip3* promoter region fragments in the enriched DNA samples.

### 2.8. Droplet Digital PCR

After dilution, the samples were subjected to Droplet Digital PCR (ddPCR) using EvaGreen Digital PCR Supermix (Bio-Rad, #1864034, Hercules, CA, USA), with the reaction system configured according to the kit’s instruction manual. Droplets were generated by adding Droplet generation oil (70 μL; Bio-Rad, #1864005, Hercules, CA, USA) to the droplet generation card and processed through the droplet generator (Bio-Rad, #1864002, Hercules, CA, USA). The droplets were then transferred to a 96-well plate for PCR reaction, following the protocol outlined in the manual. Upon completion of the PCR reaction, droplet reading was performed using the Droplet reader (Bio-Rad, #1864003, Hercules, CA, USA), and the experimental results were analyzed using the Bio-Rad QuantaSoft™ Analysis Pro (QuantaSoft AP) software version 1.4.

### 2.9. Super-Shift Electrophoretic Mobility Shift Assay (Super-Shift EMSA)

The Super-Shift Electrophoretic Mobility Shift Assay analysis was conducted using the LightShift Chemiluminescent EMSA Kit (Thermo Fisher, 20148, Waltham, MA, USA). The probes were incubated with nuclear protein, and the resultant nucleic acid-protein complexes were resolved on a 6.5% non-denaturing polyacrylamide gel. In competition experiments, a 200-fold molar excess of an unlabeled probe (Cold Probe) and a mutant probe were introduced individually. Additionally, for the super-shift experiment, an antibody targeting RORA was incorporated. Further details regarding the specific probes utilized in this study are provided in the Supplementary Materials. After electrophoresis, the probe was transferred onto a nylon membrane for subsequent analysis.

### 2.10. Statistics

Each experiment included at least three biological replicates, and a one-way analysis of variance was used to assess statistical significance. All statistical analyses were performed using the GraphPad Prism 9.5.1 software, and the results were expressed as mean ± standard deviation; *p* < 0.05 was considered to indicate a statistically significant result.

## 3. Results

### 3.1. Activation of RORA Downregulates the Transcription Level of the Bnip3 Gene in HFSCs

**Figure 1:**
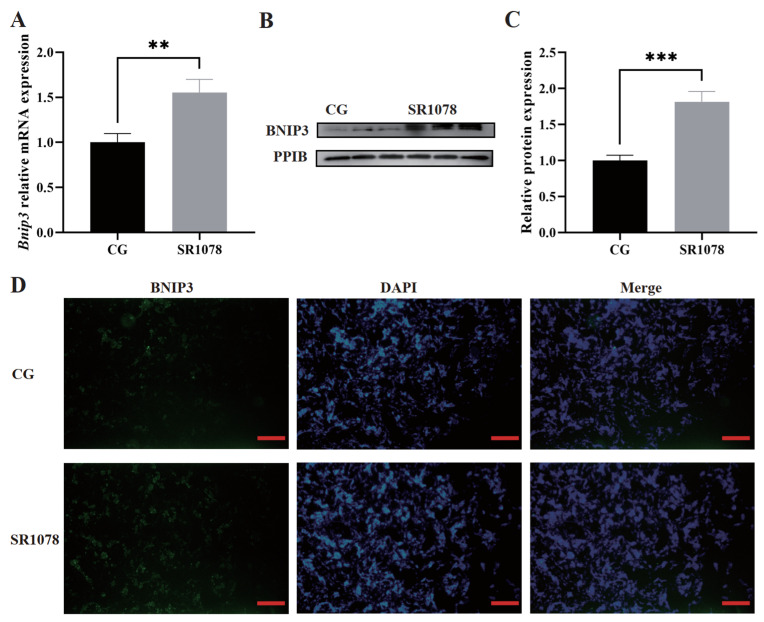
**The upregulation of *Bnip3* gene expression level by RORA activation induced by SR1078.** (**A**) Relative transcription level of the *Bnip3* gene. ** 0.001 < *p* < 0.01 (**B**,**C**) Relative expression level of BNIP3 protein. *** *p* < 0.001 (**D**) Immunofluorescence detection of differences in BNIP3 protein expression; red bar is 100 μm.

To investigate the impact of the RORA activation induced by SR1078 treatment on the transcription level of the *Bnip3* gene in HFSCs, we conducted a qPCR analysis. The melting curves for all genes displayed unique single peaks, confirming the absence of nonspecific amplification and validating the analytical methodology. After normalization with the reference gene, our results revealed that the SR1078-induced RORA activation significantly upregulated the transcription level of the *Bnip3* gene in HFSCs (*n* = 3). Specifically, the transcription level of *Bnip3* was increased by approximately 1.5-fold in the SR1078-treated group compared to the DMSO control group (Figure 1A). The findings indicate a potential regulatory role of RORA on *Bnip3*, suggesting that *Bnip3* may serve as a target gene of RORA, and provides preliminary insights into the molecular mechanisms underlying the regulation of *Bnip3* by RORA in HFSCs.

### 3.2. Activation of RORA Upregulates the Protein Expression Level of the Bnip3 in HFSCs

To further elucidate the regulatory relationship between RORA and *Bnip3*, we employed Western blotting to assess the *Bnip3* protein levels in HFSCs following SR1078 treatment. The results demonstrated that compared to the DMSO control group, the activation of RORA significantly upregulated the protein expression level of *Bnip3* (Figure 1B,C). This finding further supports the possibility that *Bnip3* may be a potential target gene of RORA. Additionally, we directly observed the *Bnip3* expression levels in both the SR1078-treated and DMSO control groups using immunofluorescence. The results showed that RORA activation led to a notable upregulation in *Bnip3* expression, which is consistent with the findings from qPCR and WB (Figure 1D). Our results collectively reinforce the existence of a regulatory relationship between RORA and *Bnip3*.

### 3.3. There Exists a Binding Relationship Between RORA and the Promoter Region of Bnip3

**Figure 2:**
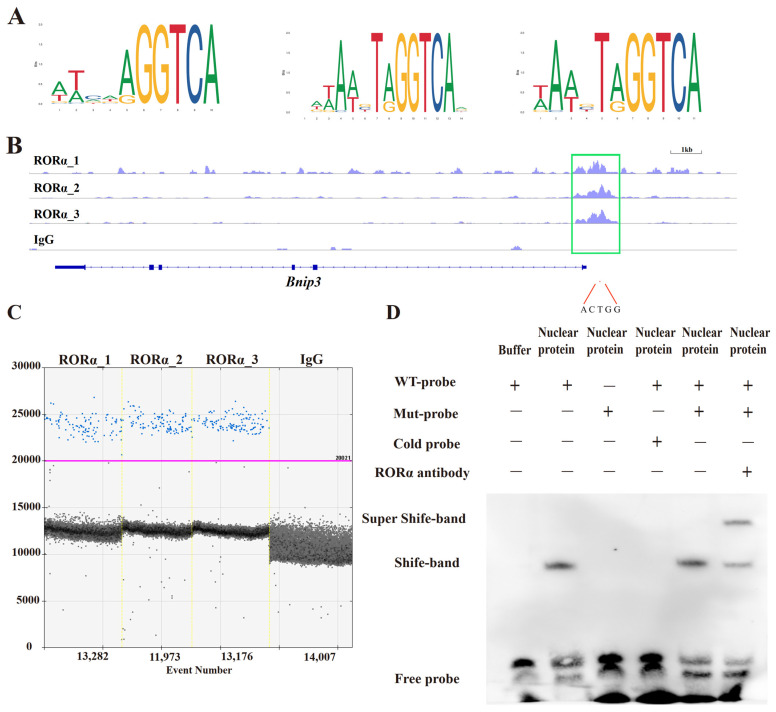
**RORA binds to the promoter region of the *Bnip3* gene to regulate its expression.** (**A**) Motif structure bound by RORA. (**B**) CUT&Tag signal in the promoter region of the *Bnip3* gene. (**C**) Fragments of the *Bnip3* promoter region were found to be present within the potential binding sites of RORA enriched by CUT&RUN through ddPCR analysis. (**D**) Super-Shift EMSA demonstrates a direct binding relationship between RORA and the promoter region of *Bnip3*.

After establishing the regulatory effect between the RORA and *Bnip3* expression, we further analyzed whether this regulation occurs directly or indirectly. In previous studies, we identified potential downstream target genes of RORA using CUT&Tag technology and observed significant peak signals in the promoter region of *Bnip3*. Using the JASPAR database and literature reports, we obtained the motifs of RORA and identified the common GGTCA sequence among these motifs (Figure 2A). Through comparative analysis, we found that the promoter region of the rat *Bnip3* gene contains this common motif sequence, and this sequence is located within the Peak of the *Bnip3* gene promoter region determined by CUT&Tag (Figure 2B). Therefore, this site is likely a direct binding site for RORA. We designed primers targeting this site and used droplet digital PCR (ddPCR) to detect the binding fragments of RORA with its downstream target genes enriched by CUT&RUN. The results were positive for the experimental group but negative for the IgG control group, which confirms the potential direct binding relationship between RORA and the promoter region of *Bnip3* (Figure 2C).

### 3.4. RORA Can Directly Bind to the Promoter Region of Bnip3

While previous CUT&Tag results and the CUT&RUN coupled with ddPCR validation in this study have demonstrated a binding relationship between RORA and the promoter region of *Bnip3*, they have not yet clarified whether this binding is direct or indirect. Therefore, we employed electrophoretic mobility shift assay technology to investigate whether RORA has the ability to directly bind to the motif sequence in the *Bnip3* promoter region (Figure 2D). The experimental results showed that the incubation of the Wt-probe with nuclear proteins produced a shift band, while the incubation of the Mut-probe with nuclear proteins did not produce a shift band. Additionally, the addition of an excess of unlabeled cold probes competitively inhibited the formation of the shift band, while an excess of Mut-probe had no such inhibitory effect. Furthermore, the addition of a specific antibody against RORA resulted in the formation of a Super-Shift band. These results provide strong evidence that the binding relationship between RORA and the promoter region of the *Bnip3* gene is direct.

## 4. Discussion

The development of animal hair is a crucial aspect in determining an animal’s ability to adapt to harsh environments, and this process exhibits a high degree of rhythmicity. It displays an intrinsic developmental cycle and is highly correlated with external factors such as the photoperiod. A typical hair follicle structure comprises dozens of cell types, each playing physiological roles in maintaining the structural integrity of the hair follicle and in the production of keratinized hair. Among these, hair follicle stem cells are important drivers of the periodic reconstruction of hair follicles. Therefore, this study used HFSCs as a research model to further analyze the potential regulatory mechanisms of hair follicle development [42,43].

RORA, as a nuclear receptor, is a typical rhythm-related gene, but our current understanding of it is still limited. Most studies have focused on related fields such as cancer and psychiatric diseases, while its impact on the development cycle of animal coat hair has been rarely reported [29,30,33,44,45]. Due to the significant correlation between the notable periodicity of hair development and the regulatory role of RORA in biological rhythms, this study attempted to analyze the interaction between RORA and its downstream target genes, further exploring its impact on the physiological state of hair follicle stem cells and its effects on the development cycle of animal hair.

*Bnip3* was initially considered to be a gene highly associated with apoptosis. *Bnip3* can form a heterodimer with the classic antiapoptotic gene *Bcl-xL* through its BH3 domain, thereby inhibiting *Bcl-xL*’s antiapoptotic ability [23,46]. Meanwhile, *Bnip3* can also form a heterodimer with *Bcl-2*, thereby blocking the antiapoptotic effect of *Bcl-2*. Additionally, when involved in apoptosis, *Bnip3* affects the changes in the mitochondrial membrane potential. An increase in its expression level decreases the potential difference across the mitochondrial membrane, thereby initiating the apoptotic process [47,48]. Furthermore, there is evidence suggesting that *Bnip3* is associated with the release of mitochondrial cytochrome C, and the release of cytochrome C further initiates a caspase cascade reaction, ultimately inducing cell apoptosis [49,50]. Current evidence also indicates a strong correlation between *Bnip3* and HIF-1α. Under hypoxic conditions, HIF-1α upregulates the levels of *Bnip3*, thereby regulating the state of the cell [51,52,53]. In addition to its strong correlation with apoptosis, *Bnip3* is also closely related to mitophagy. *Bnip3* interacts with autophagy-related proteins such as Beclin-1 and LC3, promoting the occurrence of mitophagy and regulating cellular homeostasis, but its excessive regulation of autophagy may be an important factor in the occurrence of some damage [54,55,56].

Given *Bnip3*’s crucial role in regulating physiological processes such as apoptosis and autophagy, its significance in maintaining and transitioning the physiological state of HFSCs is evident. Therefore, we analyzed the relationship between RORA and *Bnip3* and found that *Bnip3* is a downstream target gene of RORA, with its expression level directly regulated by RORA. RORA participates in this regulatory process by directly binding to the motif in the *Bnip3* promoter region. In our previous studies, we observed that the activation of RORA by SR1078 increases the apoptosis level of HFSCs, which is consistent with the results of this study. Furthermore, the increase in *Bnip3* level may be an important pathway through which RORA induces apoptosis in HFSCs.

## 5. Conclusions

Focusing on hair follicle stem cells, this study has demonstrated the regulatory effect of the nuclear receptor RORA on the expression of the *Bnip3* gene, further confirming that *Bnip3* is one of the direct target genes of RORA. RORA can upregulate the transcription level of *Bnip3* by binding to the promoter region of *Bnip3*, and this interaction may be crucial for the regulation of HFSC homeostasis. Based on our research findings, we believe that RORA can be a key target in the field of research on the regulation of seasonal hair molting in animals.

## Data Availability

The data presented in this study are available upon request from the corresponding author.

## Supplementary Materials

The following supporting information can be downloaded at https://www.mdpi.com/article/10.3390/ani14233477/s1: Supplementary Table S1. The data source file is also included in the Supplementary Files.
