# Supplementary figures and images for: Retinoic Acid-Related Orphan Receptor Alpha May Regulate the State of Hair Follicle Stem Cells by Upregulating the Expression of BNIP3
Source: Animals (Basel). 2024 Dec 2;14(23):3477. doi: 10.3390/ani14233477 (PMC11640481; doi:10.3390/ani14233477)

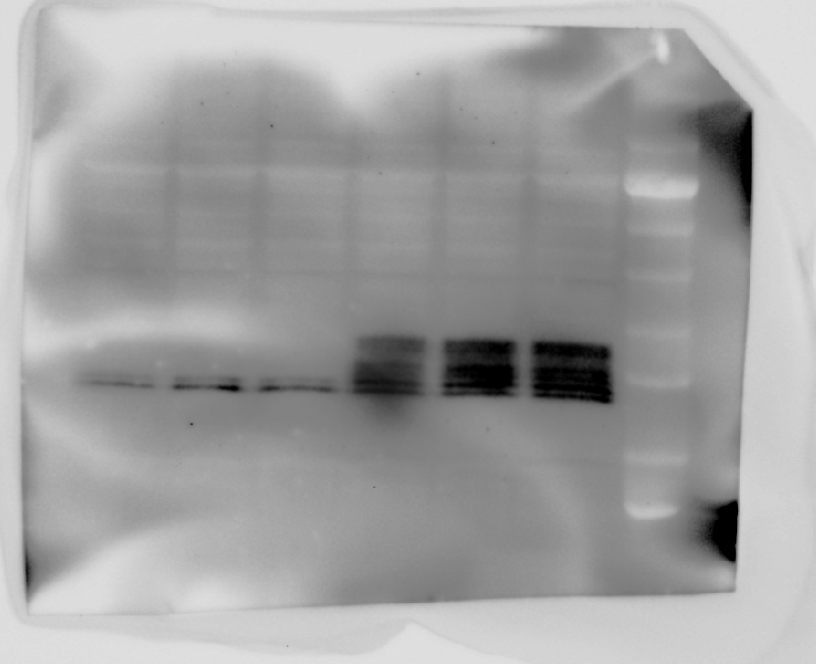

Supplement: Supplementary file 1 [file animals-14-03477-s001.zip › Uncropped image of BNIP3 of Figure 2B.tif]

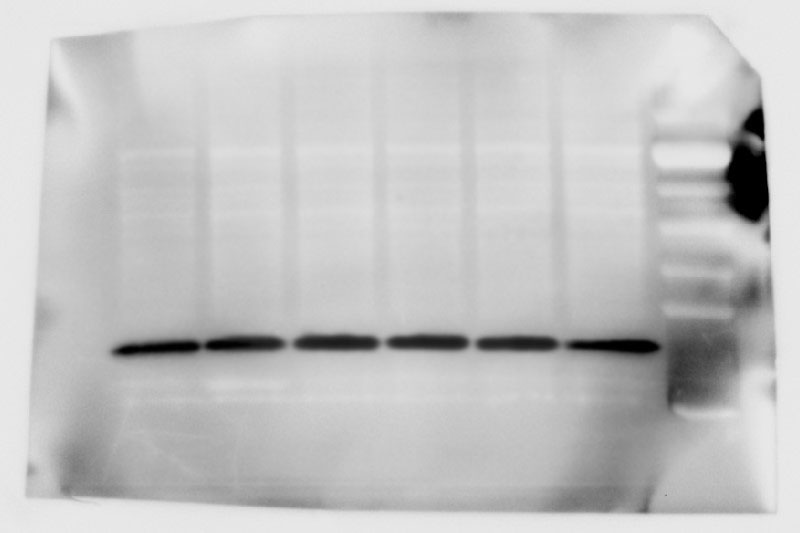

Supplement: Supplementary file 1 [file animals-14-03477-s001.zip › Uncropped image of PPIB of Figure 2B.jpg]
